# In-Process Monitoring of Lack of Fusion in Ultra-Thin Sheets Edge Welding Using Machine Vision

**DOI:** 10.3390/s18082411

**Published:** 2018-07-25

**Authors:** Yuxiang Hong, Baohua Chang, Guodong Peng, Zhang Yuan, Xiangchun Hou, Boce Xue, Dong Du

**Affiliations:** Key Laboratory for Advanced Materials Processing Technology, Ministry of Education, Department of Mechanical Engineering, Tsinghua University, Beijing 100084, China; hongyuxiang@tsinghua.edu.cn (Y.H.); bhchang@tsinghua.edu.cn (B.C.); pgd12@mails.tsinghua.edu.cn (G.P.); z-yuan15@mails.tsinghua.edu.cn (Z.Y.); houxc16@mails.tsinghua.edu.cn (X.H.); xbc17@mails.tsinghua.edu.cn (B.X.)

**Keywords:** micro plasma arc welding, edge joint weld, defects detection, weld pool monitoring, lack of fusion, micro vision sensing

## Abstract

Lack of fusion can often occur during ultra-thin sheets edge welding process, severely destroying joint quality and leading to seal failure. This paper presents a vision-based weld pool monitoring method for detecting a lack of fusion during micro plasma arc welding (MPAW) of ultra-thin sheets edge welds. A passive micro-vision sensor is developed to acquire clear images of the mesoscale weld pool under MPAW conditions, continuously and stably. Then, an image processing algorithm has been proposed to extract the characteristics of weld pool geometry from the acquired images in real time. The relations between the presence of a lack of fusion in edge weld and dynamic changes in weld pool characteristic parameters are investigated. The experimental results indicate that the abrupt changes of extracted weld pool centroid position along the weld length are highly correlated with the occurrences of lack of fusion. By using such weld pool characteristic information, the lack of fusion in MPAW of ultra-thin sheets edge welds can be detected in real time. The proposed in-process monitoring method makes the early warning possible. It also can provide feedback for real-time control and can serve as a basis for intelligent defect identification.

## 1. Introduction

Edge joint weld is a type of welding joint that is commonly used in ultra-thin-walled (thickness ≤ 0.15 mm) precision metal parts and components in industries of aircraft, aerospace, nuclear power, petrochemical, metallurgy, etc., such as the bellows-type accumulator in launch vehicle [1,2,3]. These welds are generally welded by precision welding, like micro-plasma arc welding (MPAW), micro-tungsten inert gas welding, or laser welding, which have concentrated energy distributions and narrow heat affected zones [4,5,6]. For many key welds, MPAW is preferred.

The edge welding of ultra-thin sheets is very sensitive to the changes in both heat input and heat sink. Lack of fusion, as a one of the most serious defects in edge weld, can often occur during the welding process [4,7], and severely destroy joint quality and result in seal failure. In MPAW manufacturing of ultra-thin-walled components, for instance, the aerospace accumulator bellows, the lack of fusion happens occasionally, and the repair welding rate is quite high, although the fabrication process is under strict supervision and control. So far, the understanding of this type of defect is still very limited and unsatisfactory. Several common factors are known to be in favor of the occurrence of this defect, including unstable arc, variation in heat sink, excessive thermal distortion, weld misalignment, impurities on workpiece surface, improper heat input, dimensions out of tolerance, and poor fit-up. Nevertheless, the random disturbances during welding cannot always be entirely eliminated.

At present, the defect detection for ultra-thin sheets edge welds in industry mainly rely on post-process non-destructive inspection. There is an urgent need to improve the detection efficiency and reduce the labor intensity. Taking the bellows components in aerospace accumulators as an example, the edge welds of the component are tiny and numerous, and have strict demands on weld quality and sealing property. A series of expensive, time-consuming, and labor-intensive weld inspection are carried out for the edge welds, including appearance inspection, dimension measurement, mechanical property test, hydraulic seal test, and air-tight test [7,8,9].

In-process defect monitoring/detection can indicate the presence and location of potential defects for guiding post-process inspection, and significantly improve the detection efficiency [10,11,12,13]. It is even possible to realize a real-time parameters feedback control to prevent defects. Because of the unsatisfactory stability in welding quality, it is of great necessity to achieve in-process defect detection, especially for the ultra-thin sheets edge welding.

During processes of arc welding and laser welding, various types of sources can provide online information relevant to the weld quality, such as arc voltage [14], welding current [15], audible sound [16], acoustic emissions [17,18,19], as well as the optical or thermal radiation that is generated from electric arc, molten pool, plasma plume, and metallic vapor [20,21,22,23]. A promising approach is to use machine vision to the in-process weld pool monitoring, as this provides an access to abundant and direct-viewing information about the process dynamics that closely related to weld bead formation and some defects [24,25,26,27]. Few research works have been reported in the field of MPAW regarding vision-based weld pool monitoring and defect detection. However, much research has been conducted on vision-based methods to sense the weld pool/keyhole geometry in keyhole mode plasma arc welding (K-PAW) process [28,29,30,31] and keyhole mode laser welding (K-LW) process [32,33,34,35,36,37]. The heat-transfer mechanisms of such welding processes that use keyhole mode heating are essentially different from that of MPAW process. A fully penetrated keyhole inside the weld pool is formed and full-penetration weld can be acquired during both K-PAW and K-LW. The weld pool and keyhole behaviors are the key factors to determine the welding process stability and weld joint quality in the both welding processes, thus they are of primary concern in process monitoring. Recently, a vision system has been developed to observe both the keyhole and the weld pool simultaneously from the underside of workpiece in K-PAW [28]. Through image processing and calibration, the geometric parameters of the keyhole and the weld pool are obtained. Due to the laser-material interactions that occur during the laser welding, energy is emitted in a variety of forms, and thus more status information on the welding process can be acquired by using multi-source information sensing and fusion [34,35,36]. It has been reported that a multi-optics sensing system composed of a photodiode and two vision sensors was developed to monitor the weld pool region, plasma, and spatters during high-power disk laser welding [37]. The multisensor fusion approach that is based on support vector machine was used to identify several different kinds of weld defects. In conduction mode laser welding (C-LW), the heat to create the weld occurs by conduction from the surface into the material. The weld pool is the major source of information that can be used to control the desired weld penetration in C-LW, which is relatively sensitive to the changes in heat sink [38]. There are also a number of researches conducted on process monitoring and control using weld pool visual sensing in gas tungsten arc welding (GTAW), an arc welding process that is similar to MPAW [39,40,41,42,43,44,45,46]. Coaxial vision methods were proposed and used to observe the weld pool [39,40]. In recent years, real-time measurement/reconstruction of the three-dimensional (3D) weld pool surface for GTAW process monitoring, modelling, and control has become a very active research field [43,44,45]. As the multi-sensor approach has the advantage of obtaining rich information about different welding statuses, researchers attempted to obtain the more accurate detection during the GTAW process by using the vision sensor combined with other sensors [46].

So far, the researches on weld pool visual sensing for in-process process monitoring and defect detection are mainly focused on the visualization of weld pool in a macro scale and targeted at LBW, GTAW, K-PAW, and GMAW. Few of their methods are available for the observation or monitoring of a dynamic meso-scale weld pool in strong arc lights condition. To our knowledge, far too little exploration and systematic investigation have been reported in literature on the in-process defects monitoring or detection in MPAW of ultra-thin sheets edge welds.

The primary objective of this study is to investigate the feasibility of achieving in-process monitoring of lack of fusion in MPAW of ultra-thin sheets edge welds by using machine vision approach. We focus on the following aspects:

• Micro-vision sensing of dynamic meso-scale weld pool in condition of strong arc lights

Unlike the welding process of other types of weld, an edge welding of foil-thickness metal sheets using MPAW has high intensity arc lights radiation and a very small weld pool with a diameter generally less than 0.3 mm. A fundamental and key issue to achieve effective visual sensing and reliable defect detection during MPAW of ultra-thin sheets edge weld is to build clear imaging conditions for the dynamic weld pool at the meso scale. To sense such weld pool using the vision methods requires a high magnification optical detecting technique with proper optical filtering approaches. In addition, the plasma arc length is normally kept between 0.5 and 2 mm to obtain consistent weld quality, the weld pool can only be observed at a quite small angle to weld surface due to the block and limitation by the plasma welding torch nozzle. As a result, to ensure that the entire weld pool can be imaged clearly at the meso scale, a vision system that has an enough deep depth of field (DOF) is also necessary. However, it is a complicated and difficult task to balance the conflicting demands of the weld pool visualization for high optical magnification and deep DOF of vision sensor.

• Real-time image processing and features extraction of pulsed current MPAW weld pool

The reliability and the real-time performance of defects detection heavily rely on image processing and understanding techniques. However, image quality can be easily degraded by the complex and time-varying disturbance that is introduced by the pulsed arc lights and specular reflection of weldment surface during pulsed current MPAW process. This will directly affect the accuracy, reliability, and algorithm speed of the features extraction. How to reduce these interferences and achieve characteristics extraction with an acceptable efficiency are the critical issues that need to be addressed. 

• Relation between weld pool behaviour and lack of fusion in MPAW of ultra-thin sheets edge weld

The physical processes of both arc welding and laser welding have already indicated that there is a certain correlation between optical features and geometrical feature. It is expected to predict welding quality, and especially some weld defects by establishing the mapping relation model. For the MPAW process of edge welds, the understanding of weld pool surface tension, fluid flow inside the weld pool and the mechanism causing lack of fusion are still very limited. To achieve in-process detection of lack of fusion and to provide a basis for a better knowledge of the weld pool thermo-physical behavior in the edge welding, it is essential to establish the relations between the changes in weld pool characteristics and the presence of a lack of fusion.

In this study, a passive micro-vision sensor based on object-side telecentric optical design was first developed to acquire highly magnified and enough clear images of the dynamic mesoscale molten pool during MPAW of ultra-thin sheets edge welds. Then, an image processing algorithm based on symmetric nearest neighbor filter (SNNF) and Otsu’s method was proposed to extract geometrical characteristics of molten pool from the acquired images in real time. Finally, welding experiments were carried out with 304 stainless steel diaphragms of 120 μm thick to provoke the generation of lack of fusion defects by different perturbation factors. By using the proposed visual monitoring approach and the scanning electron microscopic (SEM) analysis technique, the relations between the weld pool characteristics parameters and the lack of fusion in edge welds were investigated and a promising indicator of the lack of fusion was found. The lack of fusion in MPAW of ultra-thin sheets edge welds can be detected in real time by using such weld pool characteristic information. The proposed in-process monitoring method makes the early warning possible. It also can provide feedback for real-time control and can serve as a basis for intelligent defect identification.

## 2. Experimental Method and Setup

### 2.1. Vision Sensing of Weld Pool

**Figure 1:**
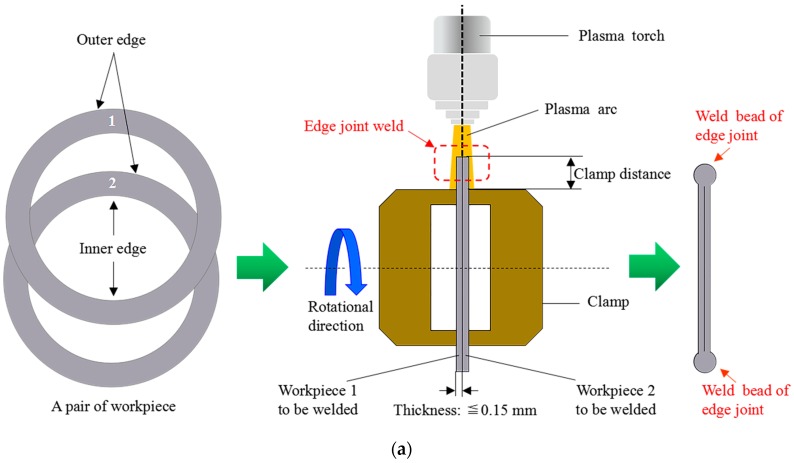

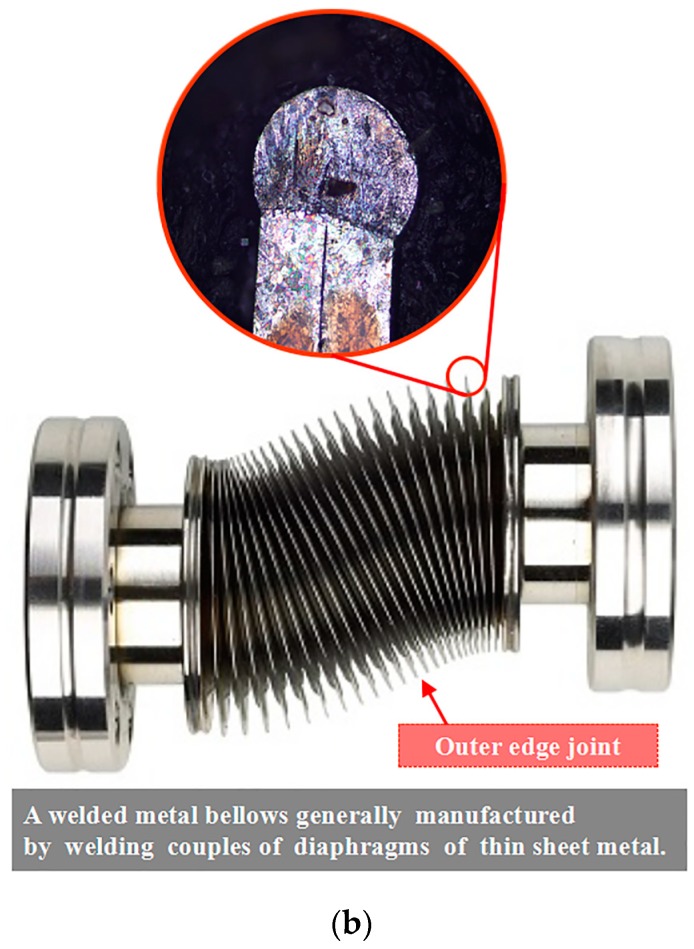
The schematic diagram for the micro-plasma arc welding of edge weld in an ultra-thin sheets edge joint: (**a**) Two-dimensional view of the edge welding; (**b**) An example of the edge-welded metal bellows using micro-plasma arc welding (MPAW).

As shown in Figure 1, an edge joint weld differs from other types of weld, such as fillet, vee, lap, and butt welds. To made an edges weld, two parallel metal sheets less than 0.15 mm thick are set side by side and welded together at the edges. Clamp distances of 1 to 4 times the thickness of the sheets are adopted to ensure the correct joint fit-up and prevent warping. An example of the edge-welded metal bellows using MPAW is shown in Figure 1b. The robust vision sensing of the dynamic mesoscale weld pool in MPAW of ultra-thin sheets edge weld places high demands on spatial resolution, definition, and signal-to-noise ratio of the captured images. A specially designed micro-vision sensor is therefore required. 

**Figure 2:**
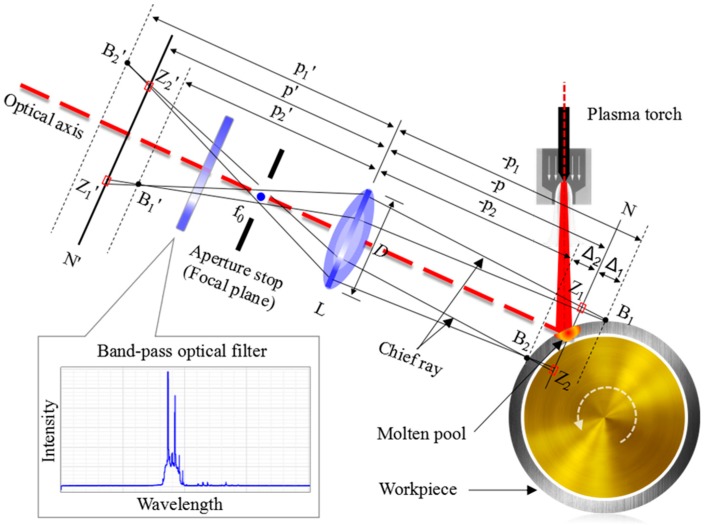
Image formation for dynamic mesoscale weld pool in MPAW using an object-side telecentric optical model.

In our micro-vision sensor, a design that is based on object-side telecentric optical model [47] is used. Figure 2 illustrates the schematic diagram of image formation for dynamic mesoscale weld pool in MPAW using object-side telecentric optical model. Let *L* denotes the magnification lens, and *D* stands for diameter of the entrance pupil. Object point *B*_1_ and *B*_2_ are in the two sides of object plane *N* separately, and the image point *B*_1_*’* and *B*_2_*’* are also in the two sides of image plane *N’*. Object distance and image distance are, respectively, *p* and −*p*. The projection of *B*_1_ on *N’* is circle of confusion *Z*_1_*’*, and the projection of *B*_2_ on *N’* is circle of confusion *Z*_2_*’*. The *Z*_1_ and *Z*_2_ are conjugate sections of the *Z*_1_*’* and *Z*_2_*’,* respectively. Unlike the commonly used pinhole model in machine vision, a small aperture stop is placed at the image-side focal point of the magnification lens *f*_0_, thus only the light rays parallel to the optical axis from the object side are allowed to pass through the aperture stop and form the image on *N’*. Ignoring the distortion, this imaging model can be expressed by

(1) [uv1]=[εΔu−ε⋅cotθΔv00εΔv⋅sinθ0001][r11r12r13txr21r22r23ty0001][XWYWZW1] 

where [*X_W_*, *Y_W_*, *Z_W_*]^T^ is the coordinate of the object point *P* in the world coordinate system {*W*}. [*u*, *v*]*^T^* is the coordinate of *P’s* image point in pixel coordinate system {*P*}. *ε* is the effective magnification of the lens. Δ*u* and Δ*v* are the unit metric lengths of a pixel in the *u* and *v* directions, respectively. *θ* is the skew angle of the imaging sensor pixel array. Denote *^C^R_W_* and *^C^t_W_* as the rotation matrix and the translation vector of the transformation from the world coordinate system {*W*} to the camera coordinate system {*C*}, respectively, the [*r*_11_, *r*_12_, *r*_13_] and [*r*_21_, *r*_22_, *r*_23_] are the first two rows of the rotation matrix *^C^R_W_*, and *t_x_* and *t_y_* are the first two elements of *^C^t_W_*.

In this model, the aperture stop setting allows for a smaller aperture angle of light rays to emerge from the object side and a smaller circle of confusion to be formed on image plane. Therefore, the shallow DOF (namely Δ_1_ + Δ_2_) introduced by high optical magnification lens can be improved somewhat. The entrance pupil is located at infinity on the object side, and an orthographic projection of objects in the world space is produced. This solves the problem of image magnification variation with object distance, which cannot be ignored in the precision detection of micro objects. The band-pass optical filter is used to reduce the interference from the strong arc light during the MPAW process, for obtaining images of weld pool region with enough signal-to-noise ratio.

**Figure 3:**
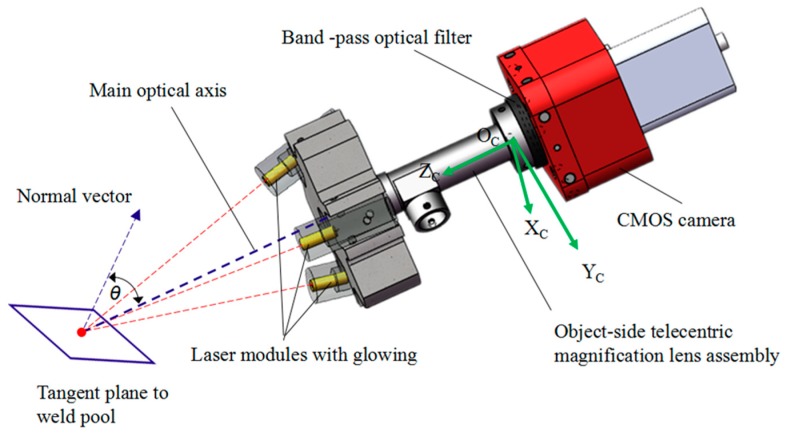
The configuration of the designed passive micro-vision sensing sensor.

**Figure 4:**
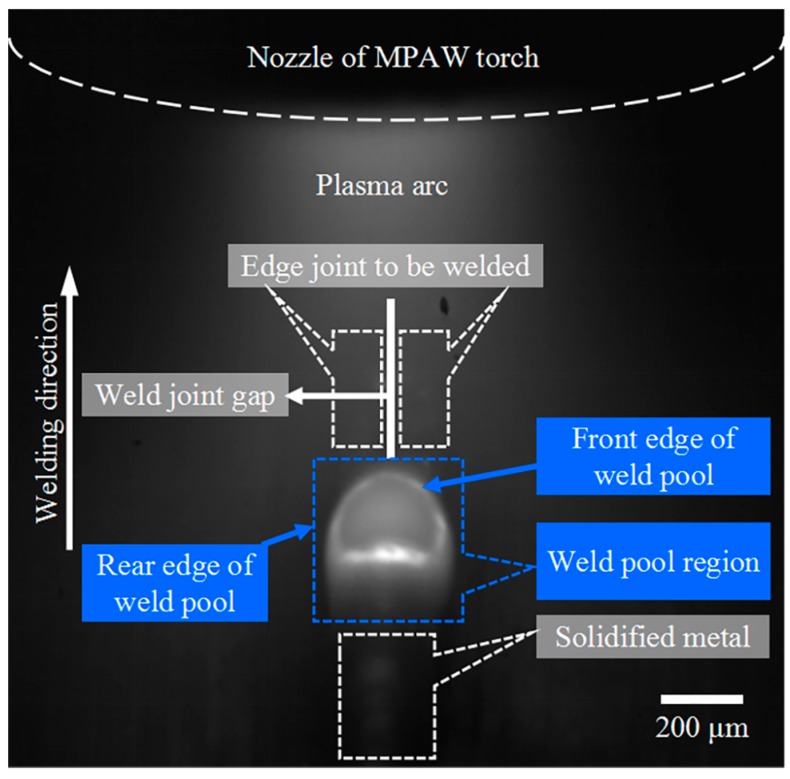
The top view of a typical weld pool in MPAW of edge weld with 0.12 mm-thick sheets.

As shown in Figure 3, the designed passive micro-vision sensor is composed of a CMOS camera, the object-side telecentric magnification lens assembly, a band-pass optical filter, and the laser modules with glowing. The camera is Photonfocus MV1-D1024E-160-CL with a maximum frame rate of 150 fps at maximum size of 1024 × 1024 pixels, which is mounted with the object-side telecentric lens assembly featuring 2× optical magnification and 0.031 numerical aperture. The band-pass optical filter is placed between the lens assembly and the CMOS camera. The central wavelength and the full width at half maximum (FWHM) of the bandwidth filter are 635 nm and 10 nm, respectively. The laser modules with glowing used for precise manual focusing before welding is arranged on the front end of the lens. The sensor has a 2.66 mm × 3.59 mm field of view and a 0.64 mm depth of field. It is mounted on a linear stage with a determined angle to focus on the features of interest. The resolution of the images is approximately 6 μm by calibration. Such a design of the micro-vision sensor allows for good quality imaging of dynamic molten pool in MPAW. A typical weld pool image captured from proposed micro-vision sensor during MPAW process of edge weld with 0.12 mm-thick sheets is shown in Figure 4. In this case, the frame rate of camera was set at 175 fps.

### 2.2. Features Extraction of Weld Pool by Image Processing

**Figure 5:**
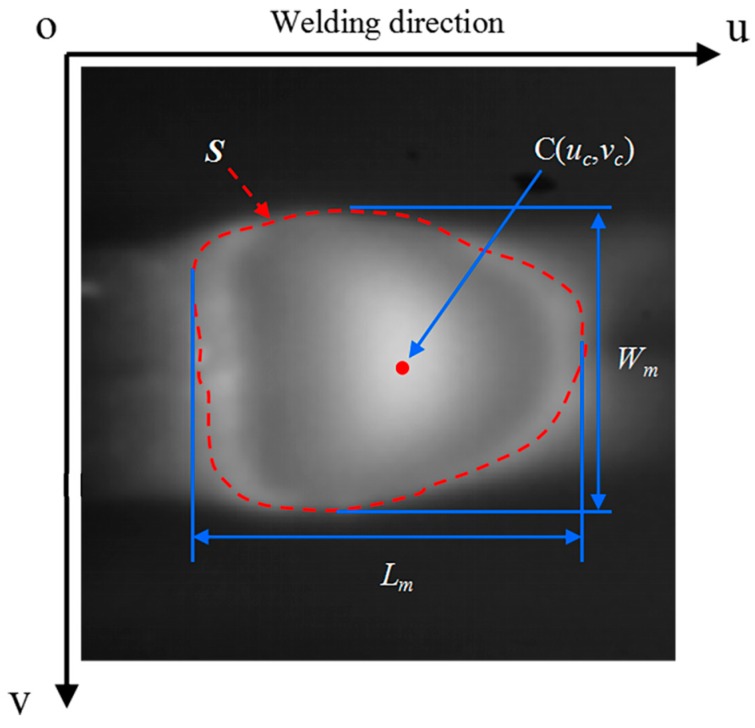
Definition of characteristic parameters of a weld pool.

As shown is Figure 5, the UV-coordinate system is pixel coordinate system {*P*} of the proposed micro-vision sensor. *S* are defined as weld pool, and three parameters: *W_m_*, *L_m_*, and *C*(*u_c_,v_c_*) are defined as the characteristic parameters of weld pool. Particularly, parameter *W_m_* and Parameter *L_m_* represents the maximum width and length of the weld pool, respectively. Parameter *C*(*u_c_,v_c_*) represents the centroid coordinates of the weld pool in the pixel coordinate system {*P*}. 

A feature extraction algorithm for images that are captured by the above sensing method was proposed to detect the edges and geometrical characteristic parameters of weld pools. The feature extraction algorithm was written in C++ and OpenCV library. Denote the captured original image as *I*_1_(*u*,*v*). The steps of the algorithm are as follows:

1. Selection of the region of interest (ROI). The sums of intensity values in sliding windows within the image *I*_1_(*u*,*v*) are calculated. The window that has the maximum sum is selected as the ROI, so that it can include the entire weld pool region. The ROI can help to exclude part of arc and spatter as well as reduce the computational effort. Denote the ROI as *R*_1_(*u*,*v*).
2. Contrast stretching. The operation saturates the bottom 1% and the top 1% of all pixel values, and it maps other pixel values linearly.
3. Noise reduction with the SNNF. The SNNF [48] is a two-dimensional (2D) nonlinear filter that reduces noise while at the same time preserving edge content. This algorithm uses both spatial and nearest-neighbor constraints on image pixels to smooth an image. It is simple, fast, and good at preserving weld pool contour in images. The image after noise reduction is denoted as *N*_1_(*u*,*v*).
4. Image segmentation with Otsu’s method. Otsu’s method [49] calculates the optimum threshold separating the image into the foreground and the background so that their intra-class variance is minimal. After image segmentation, the weld pool belongs to the foreground.
5. Search of the maximum connected domain, namely *S*. As a result, the weld pool can be identified.
6. Extraction of the weld pool contour and features.

The parameter *W_m_* can be calculated by:

(2) $$W_{m}=\underset{(u,v)\in S}{\max}v-\underset{(u,v)\in S}{\min}v\;$$

where *u* and *v* denote horizontal coordinate and vertical coordinate in pixel coordinate system, respectively.

The parameter *L_m_* can be calculated by:

(3) $$L_{m}=\underset{(u,v)\in S}{\max}u-\underset{(u,v)\in S}{\min}u\;$$

The parameter *u_c_* and *v_c_*, namely centroid coordinates, can be figured out by:

(4) $$uc=\frac{\sum_{(u,v)\in S}u}{A},yc=\frac{\sum_{(u,v)\in S}v}{A}\;$$

where *A* is the number of pixels in region *S*, which represents the area of weld pool.

**Figure 6:**
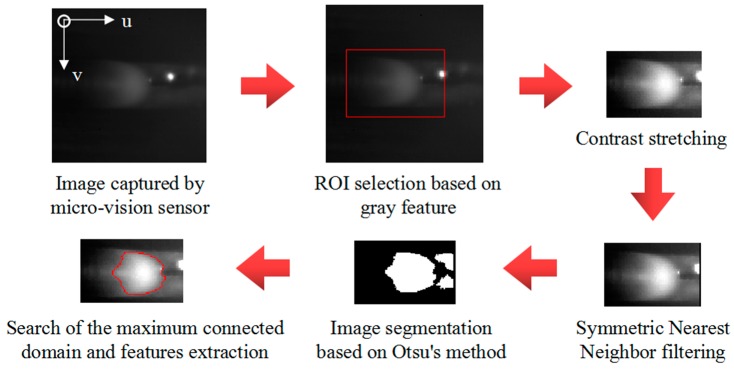
The processing result of each step of the proposed features extraction algorithm.

Figure 6 presents the processing result of each step. This algorithm is used to process over 100,000 test images that were acquired under several different welding conditions. The results show that the time cost of the algorithm is less than 10 ms per frame. 

### 2.3. Experiment System

**Figure 7:**
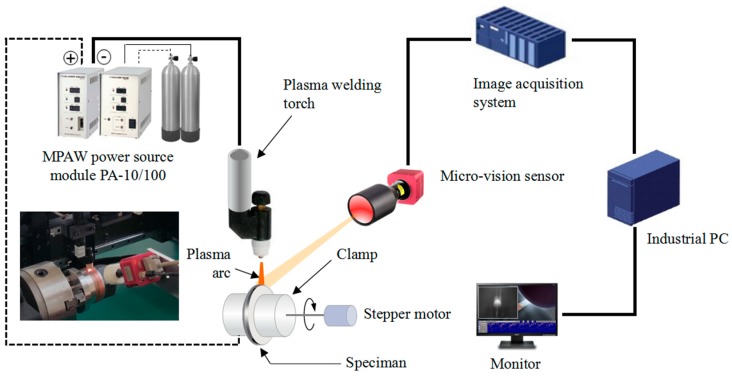
The schematic diagram of micro plasma arc welding experimental apparatus.

The schematic diagram of micro plasma arc welding experimental system is shown in Figure 7. The system mainly comprises a MPAW power source, the micro-vision sensor, an image acquisition system, and an industrial computer. The MPAW power source consists of a Weldlogic PA-10/100 micro arc welding machine, a Weldlogic PT-10 plasma welding console, a Thermal Dynamics PWH/M-2A plasma welding torch, and a water-cooled controller. The industrial computer is responsible for image processing, signal analysis, and man-machine interface. The CPU frequency and RAM size of the industrial computer are 2.3 GHz and 4 GB, respectively. The workpieces are coupled 304 austenitic stainless steel diaphragms with dimensions of an external diameter 81 mm, an inner diameter 66 mm, and thickness 0.12 mm, which are clamped on the rotary table driven by a precise stepper motor. The experimental conditions are shown in Table 1. Experiments under different welding conditions were conducted to investigate the relations between the presence of lack of fusion in stainless steel edge welds and dynamic changes in weld pool characteristic parameters. Several common perturbation factors in practical edge welding, including surface contamination, variation in the joint gap width, and weld misalignment, were artificially introduced under controlled conditions to simulate the unstable edge welding processes. These disturbances are hard to be completely eliminated in continuous industrial welding production, even for the MPAW manufacturing process of key spacecraft component that is under strict supervision and control. The relationship between the variations in weld pool characteristic parameters and the lack of fusion was investigated by means of SEM analysis technique, which was used to locate the defects and to observe the macro-morphology of the defective welds. The experiments also can verify the feasibility of achieving in-process monitoring of lack of fusion during MPAW of ultra-thin sheets edge welds by the proposed micro-vision sensing and image processing techniques. 

## 3. Experimental Results and Discussion

**Figure 8:**
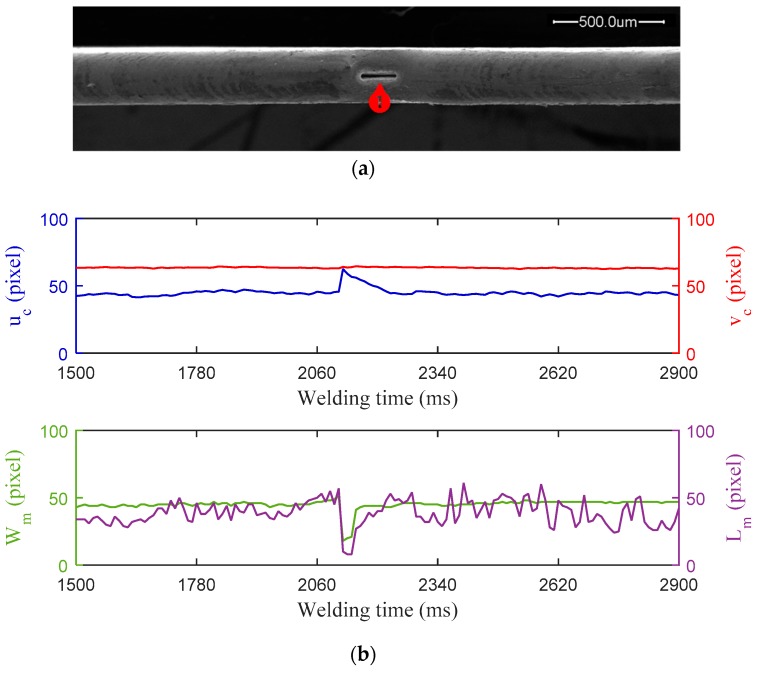
Experiment results with lack of fusion caused by surface contamination on workpiece: (**a**) Top-view of the edge weld profile observed under scanning electron microscopic (SEM) with 100× magnification; (**b**) Characteristic parameters of weld pool monitored during MPAW.

During practical ultra-thin sheets edge welding, impurities or oxides existing on the pool surface can cause unstable arc and significantly affect the surface tension of melted joint edges, and as a result give rise to the lack of fusion. Careful cleaning procedures to remove surface contamination on workpiece before welding is necessary to decrease the occurrence of such impurities and oxides. In the first experiment, the workpiece was not cleaned before welding in order to obtain a surface-contaminated edge weld. Figure 8 shows the experiment results that were obtained when the lack of fusion was introduced by surface contamination on workpiece, which include the SEM images of the edge weld profile and the synchronous monitoring results of the weld pool characteristic parameters during MPAW. The peak current, base current, and pulse frequency of the welding current are 4.80 A, 2.40 A, and 80 Hz, respectively. The welding speed is 3.65 mm/s. The exposure time of the vision sensor is 8 ms and the frame rate is 104 fps. As shown in Figure 8a, the defect of lack of fusion is produced in the middle section of the weld bead. The characteristic parameters *W_m_*, *L_m_,* and *u_c_* show their obvious sensitivity to the presence of this defect respectively. Particularly, the curve of *u_c_* showed a significant change during the same period of time, rising sharply to reach a peak value, and then return back to the value close to normal gradually*.* There are no conspicuous variations but smooth fluctuations in the curve of *v_c_*. The curves of *W_m_* and *L_m_* present similar trends of fluctuation where the defect occurred, which decline rapidly first and then return gradually. It is also observed that the curve of characteristic parameter *L_m_* fluctuates more severely than other parameter curves during the whole welding process, which can probably ascribed to the fact that its feature extraction is disturbed by the time-varying arc light reflected by solidified weld metal at the rear of molten pool.

**Figure 9:**
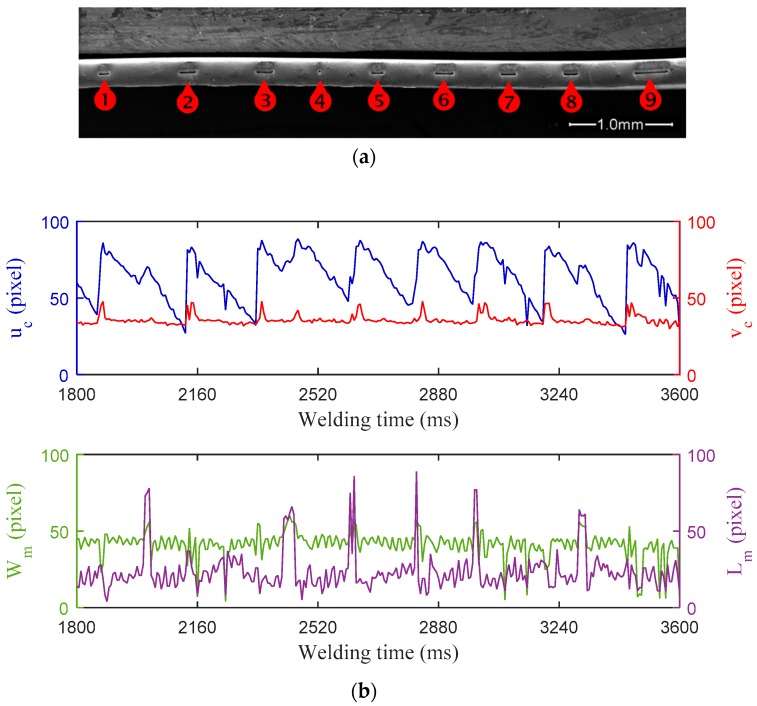
Experiment results with lack of fusion caused by excessive weld joint gap: (**a**) Top-view of the edge weld profile observed under SEM with 46× magnification; and, (**b**) Characteristic parameters of weld pool monitored during MPAW.

In the edge welding of a 0.12 mm thick aerospace accumulator bellow, a maximum allowable joint gap width is 0.024 mm to ensure that both joint edges are in good contact and that both edges melt simultaneously and fuse together into a smooth fusion weld. In addition, a fit-up tolerance of 20% the sheet thickness is also required in order to prevent warping of the joint. Even so, variations in joint gap width may happen for some unexpected disturbances and lead to lack of fusion in succession, which need to be detected in early time. In the second experiment, a decreased fit-up accuracy was used to cause joint gap variations. Figure 9 shows the experiment results when the lack of fusion was introduced by variation in the joint gap width. The variation in gap width resulted from a fit-up with excessive tolerance (0.05 mm), leading to lack of fusion in the edge weld. The mechanisms behind it are the thermal distortion of workpiece and the arc deflection. The peak current, base current, and pulse frequency of the welding current are 4.30 A, 2.15 A, and 50 Hz, respectively. The welding speed is 3.65 mm/s. The exposure time is 4 ms and the frame rate is 176 fps. As can be seen from the Figure 9a, nine defects of lack of fusion are distributed along the welding path of the weld bead. The widths of these defects are about 15 μm and the lengths of defects range from 15 μm to 300 μm. It can be observed that the characteristic parameters *u_c_* and *v_c_* are highly sensitive to the occurrence of these defects of lack of infusion. The curve of *u_c_* presents obvious fluctuations that rise firstly and then fall during the formation of each defect. It is noteworthy that a similar trend of variation, with smaller peak value, was also observed in the curve of *v_c_*. This result may be related to the perturbation on the plasma arc caused by the changing weld joint gap due to the excessive fit-up tolerance. The monitoring results indicate that the curves of *W_m_* and *L_m_* present some abnormal and irregular variations during the whole welding process, respectively, but the significant abrupt changes or singularities in both feature curves do not necessarily correspond in time domain with the presence of these defects.

**Figure 10:**
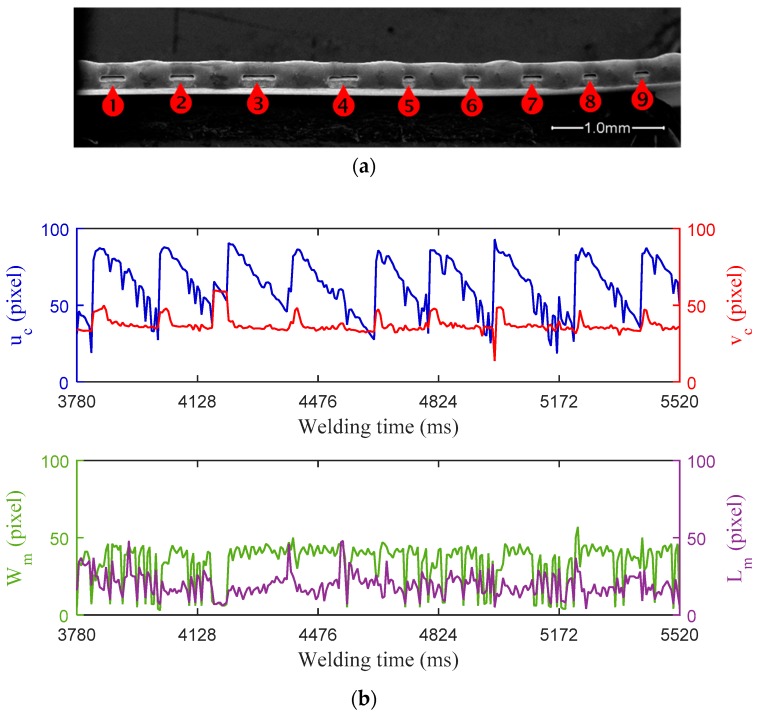
Experiment results with lack of fusion caused by weld misalignment: (**a**) Top-view of the edge weld profile observed under SEM with 50× magnification; (**b**) Characteristic parameters of weld pool monitored during MPAW.

High positional accuracy is needed in MPAW of ultra-thin sheets edge weld, but in the continuous industrial manufacturing process it is quite difficult to position the workpieces with sufficient accuracy for every weld. A small offset can result in lack of fusion because the edge welding has a low tolerance to the asymmetry of thermal distribution in the workpieces. In the third experiment, the lateral offset between the torch axis and the weld seam center are set to 0.3 mm before welding by adjusting the rotary table position. Figure 10 shows the experiment results when lack of fusion introduced by weld misalignment. The peak current, base current, and pulse frequency of the welding current are 4.60 A, 2.30 A, and 50 Hz, respectively. The welding speed is 3.65 mm/s. The exposure time is 4 ms and the frame rate is 176 fps. As can be seen from the Figure 10a, there are nine defects of lack of fusion that are distributed along the welding path of the weld bead. The width of weld bead is obvious nonuniform, which might be caused by asymmetric temperature distribution of the two workpiece. It can be observed from Figure 10b that the feature curve of *u_c_* demonstrates the best performance in indicating the presence of lack of fusion. The curve of *v_c_* also presents a strong relationship with these defects, though a few of its peaks are relatively small. This phenomenon indicates that a fluctuation of the weld pool position in the direction vertical to weld seam has taken place in the generation of each defect, which can be attributed to the arc instability caused by weld misalignment. Especially, there are only few correspondences between the significant changes in the curve of *W_m_* or *L_m_* and the occurrence of these defects. The reason for this is not clear, but it may have something to do with the instability of molten pool intensified by the asymmetry thermal distribution on the workpieces or the process disturbance from molten metal flow around the neighbouring defect.

The Steenbeck’s minimum principle (SMP) is widely used in the study of arc discharge plasmas, and it can be broadly understood as follows: The arc discharge steadily with a column radius that the axial electric field in the arc attains the minimum value under the given current and boundary conditions. It means that any change in the radius resulting from external interference will leads to a unstable arc, and the unstable arc will vary in a way that the axial electric field in the arc attains a minimum value. This principle has been proved by numerous practices and extensively invoked in investigations on many gas discharge phenomena, in particular, cylindrical arcs.

**Figure 11:**
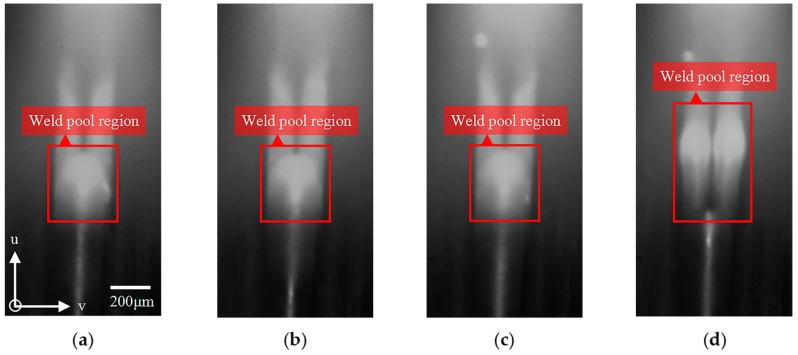

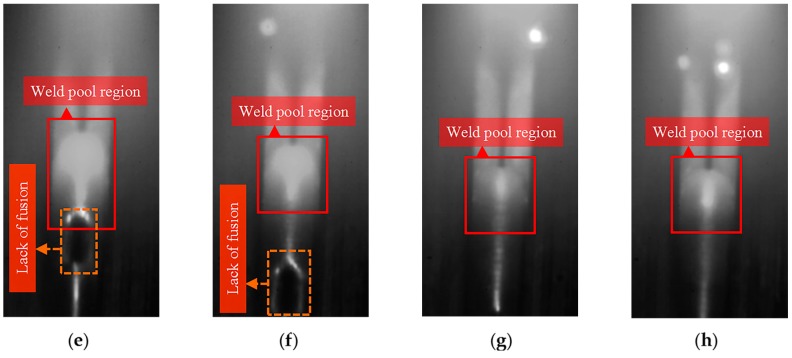
A typical weld pool behavior during the formation of lack of fusion in MPAW of edge weld. (**a**) t_0_; (**b**) t_0_ + 80 ms; (**c**) t_0_ + 160 ms; (**d**) t_0_ + 240 ms; (**e**) t_0_ + 320 ms; (**f**) t_0_ + 400 ms; (**g**) t_0_ + 480 ms; and, (**h**) t_0_ + 560 ms.

As can be seen from the above results, the fluctuation in characteristic parameter *u_c_* has exhibited obvious coincidence with the presence of lack of infusion. It indicates that the lack of fusion caused by these different physical origins is all related to the fluctuations in weld pool position, which result from the deflection of plasma arc during the welding processes. A possible explanation for this complex physical process is that the surface contamination, variation in the joint gap width, and weld misalignment can all lead to a disturbed plasma arc. Due to the arc discharge mechanism based on SMP, the disturbed plasma arc is deflected along the welding direction from its normal path and gives rise to the great fluctuation in weld pool position. The edge weld located underneath the plasma torch cannot be uniformly heated so that the weld fails to correctly melt and solidify. As a result, the lack of fusion is formed. A typical example of the dynamic change of weld pool during the formation of lack of fusion can be intuitively and clearly seen from the Figure 11. In this example, a defect of lack of fusion in edge weld can be readily observed through the proposed micro-vision sensor due to its relative large size. It can be seen that the weld pool position fluctuated along the length of weld during the formation process of the lack of fusion. Moreover, the weld pool divided into two small parts before the occurrence of a lack of fusion, and then incorporated after the defect occurred.

**Figure 12:**
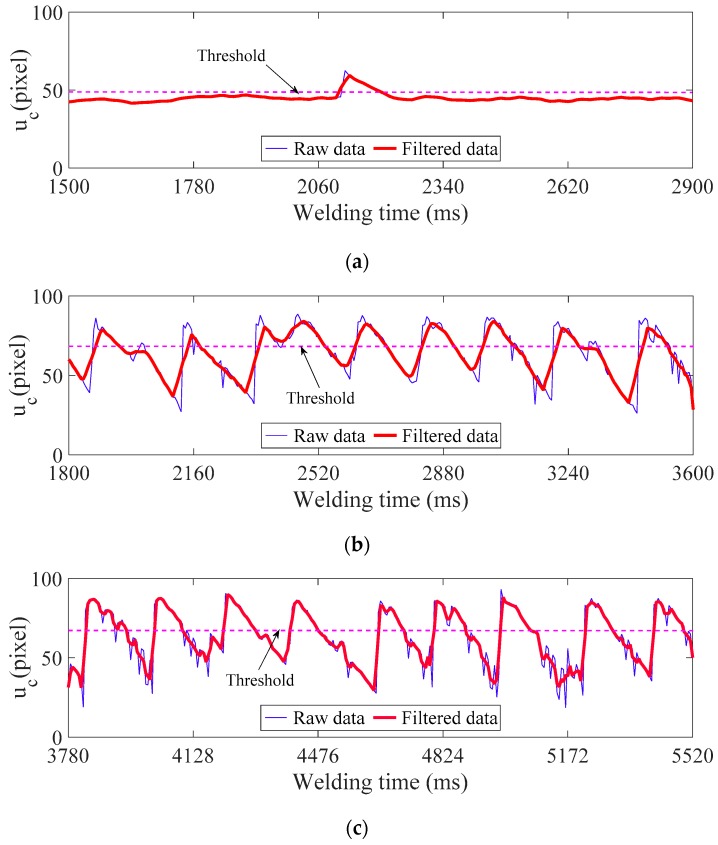
Detection results using standard deviation (STD) threshold in monitoring parameters *u_c_*: (**a**) *u_c_* in Figure 8; (**b**) *u_c_* in Figure 9; and, (**c**) *u_c_* in Figure 10.

Figure 12 shows the detection results of above experiments using the standard deviation threshold method (STDM) in monitoring parameter *u_c_*. The reference signals have been previously monitored and recorded during the welding processes with sound welds, respectively. Both the monitoring signals and the reference signals were filtered by using the simple moving average method to reduce noise. The corresponding thresholds are given by:

(5) $$Threshold=mean_{ref}+\sigma STD_{ref}\;$$

where σ is the coefficient of threshold scope and is set as 4 in all of the experiments in this paper, $mean_{ref}$ and $STD_{ref}$ are mean value and standard deviation value of *M* smoothed reference signal samples, respectively, which are calculated by:

(6) $$\{\begin{array}{c} mean_{ref}=\left(\sum_{m=1}^{M}R(m)\right)/M\\ STD_{ref}=\sqrt{\frac{\sum_{m=1}^{M}{\left[R(m)-\left(\sum_{m=1}^{M}R(m)\right)/M\right]}^{2}}{M}} \end{array}\;$$

where *m* = 1, 2, …, *M* is sampling number and R(m) refers to *m*-th sampling values.

It can be observed that all of the defects formed in these welding processes were detected based on STDM, as the monitoring signal exceeded the established threshold value where each defect presence was verified. 

By adjusting the value of σ, the threshold value is changed, which makes the monitoring system more or less sensitive to variations of characteristic parameter *u_c_*. The lower the σ-values, the more sensitive is the defect detection. Specially, the system can detect defects of lack of fusion with high certainty when σ-values are between 2 and 5. As a monitoring signal beyond the threshold for a short time does not necessarily imply the formation of lack of fusion, the defect detection algorithm will not flag a weld as defective when only *k* of *K* consecutive sample points are out of tolerance. Thus, in practical applications, the sensitivity of our monitoring system to defects detection can be modified by operating three parameters: *k*, *K,* and σ, according to the actual project needs.

In summary, the results of this study indicate that the characteristic parameters *W_m_*, *L_m_*, *u_c_*, and *v_c_* have different degrees of sensitivity to lack of fusion. The great fluctuation in *u*-coordinate of weld pool centroid (*u_c_*), which stands for an abrupt change of weld pool centroid position along weld length, was considered to be the most favourable indication for the presence of lack of fusion. The proposed in-process monitoring method using machine vision provides a powerful means of detecting lack of fusion in ultra-thin sheets edge welds during the MPAW process, which makes the early warning possible, and it can provide feedback for real-time control and serve as a basis for intelligent defect identification. Further research should be undertaken to the further development of the micro-vision system and its applications in detecting other weld defects and imperfections occurred in ultra-thin sheets edge welding, like humping weld or uneven weld bead. Also, more systematic process monitoring approaches need to be investigated by means of combining machine vision with other sensing techniques, like arc sensing and multi-spectral sensing, which would be helpful in defects identification and better understanding of defect formation mechanisms.

## 4. Conclusions

This paper proposed an effective vision-based weld pool monitoring method for detecting lack of fusion during MPAW of ultra-thin sheets edge welds. The main conclusions are summarized, as follows:

(1) The developed micro-vision sensing system can overcome the strong arc disturbance and the trade-off between optical magnification and depth of field. Thus, the morphology of mesoscale weld pool and its tiny dynamic variations can be successfully observed and stably monitored in MPAW of ultra-thin sheets edge welds, which are crucial for reliable process monitoring and defects detection. The resolution of the images is 6 μm/pixel by camera calibration.
(2) The proposed image processing algorithm based on SNNF and Otsu’s method is applicable to effectively extract geometrical features from the acquired weld pool images, e.g., maximum width, maximum length, and centroid position of weld pool, which have close relationship with the weld bead formation and defects. The processing time is less than 10 ms per frame, which is enough to satisfy the demands for monitoring and detection in real time.
(3) The variations in extracted characteristic parameters during MPAW process show various degrees of sensitivity to the weld defect of lack of fusion. Particularly, the change in weld pool centroid position along weld length is found to be a promising indicator of the lack of fusion, as the fluctuation in *u*-coordinate of weld pool centroid (*u_c_*) shows obvious coincidence with the occurrence of the defects caused by various factors.
(4) By using the characteristic parameters *u_c_*, the presences of lack of fusion defects in MPAW of ultra-thin sheets edge welds can be detected in real time. The proposed in-process monitoring method makes the early warning possible. It also can provide feedback for real-time control and can serve as a basis for the intelligent defect identification. It is expected to be applied in edge welding of precision metal parts and components in the industry of aircraft, aerospace, nuclear power, petrochemical, etc., and it is especially suitable for the welded bellows and micro pipelines.

## Figures and Tables

**Table 1:** Experimental condition of MPAW for 304 stainless steel diaphragms.

Parameter	Value	Parameter	Value
Welding materials	304 stainless steel diaphragms	Shielding gas	99.99% pure argon
Type of welding seam	Edge joint	Shielding gas flow rate	20.0 SCFH
Peak current	3.0–5.0 A	Plasma gas	99.99% pure argon
Base current	1.5–2.5 A	Plasma gas flow rate	0.4 L/min
Pulse rate	0–100 pps	Clamp distance	0.25 mm
pulse width	50%	CTWD	1.0 mm
Travel speed	3.65–13.15 mm/s	Electrode diameter	1.0 mm

## References

[1] Larsen C.E. NASA Experience with pogo in human spaceflight vehicles. Proceedings of the NATO RTO Symposium ATV-152 on Limit-Cycle Oscillations and Other Amplitude-Limited, Self-Excited Vibrations.

[2] Zhang Z., Wang N., Liu Z.S. (2014). POGO reduction technology of Chinese manned launch vehicles. Sci. Sin..

[3] Wang X.J., Yu Z.W., Zhang B., Zhang Q.S., Pan H. (2014). Progress of POGO suppression technology of launch vehicles at home and abroad. Sci. Sin..

[4] Tseng K.H., Hsieh S.T., Tseng C.C. (2003). Effect of process parameters of micro-plasma arc welding on morphology and quality in stainless steel edge joint welds. Sci. Technol. Weld. Join..

[5] Prasad K.S., Chalamalasetti S.R., Damera N.R. (2015). Application of grey relational analysis for optimizing weld bead geometry parameters of pulsed current micro plasma arc welded inconel 625 sheets. Int. J. Adv. Manuf. Technol..

[6] Batool S., Khan M., Jaffery S.H.I., Khan A., Mubashar A., Ali L., Khan N., Anwar M.N. (2015). Analysis of weld characteristics of micro-plasma arc welding and tungsten inert gas welding of thin stainless steel (304L) sheet. J. Mater. Des. Appl..

[7] Ren M., Wang C., Tian X., Zhao J. (2012). Micro-plasma arc welding manufacturing technology of bellows. Weld. Join..

[8] Park M.C., Son J.Y. 3D display and image processing system for metal bellows welding. Proceedings of the Conference on Three-Dimensional Imaging, Visualization, and Display 2010 and Display Technologies and Applications for Defence, Security, and Avionics.

[9] Park M.C., Byun Y.T., Kim D.W. (2015). Use of support vector machines for defect detection in metal bellows welding. J. Korea Soc. Comput. Inf..

[10] Norman P., Engström H., Kaplan A.F.H. State-of-the-art of monitoring and imaging of laser welding defects. Proceedings of the 11th NOLAMP Conference in Laser Processing of Materials.

[11] Zou Y., Du D., Chang B., Ji L., Pan J. (2015). Automatic weld defect detection method based on Kalman filtering for real-time radiographic inspection of spiral pipe. NDT E Int..

[12] Shao J., Du D., Chang B., Shi H. (2012). Automatic weld defect detection based on potential defect tracking in real-time radiographic image sequence. NDT E Int..

[13] Shao J., Du D., Chang B., Shi H. (2011). Automatic weld recognition and extraction from real-time X-ray images using quadratic curve fitting and multi-order differences analysis of intensity profile. Insight.

[14] Zhang Z., Chen X., Chen H., Zhong J., Chen S. (2014). Online welding quality monitoring based on feature extraction of arc voltage signal. Int. J. Adv. Manuf. Technol..

[15] Chen B., Feng J. (2014). Multisensor information fusion of pulsed GTAW based on improved D-S evidence theory. Int. J. Adv. Manuf. Technol..

[16] Horvat J., Prezelj J., Polajnar I., Čudina M. (2011). Monitoring gas metal arc welding process by using audible sound signal. Stroj. Vestnik-J. Mech. Eng..

[17] Cocota J.A.N., Garcia G.C., Costa A.R.D., Lima M.S.F.D., Rocha F.A.S., Freitas G.M. (2017). Discontinuity detection in the shield metal arc welding process. Sensors.

[18] Alfaro S.C.A., Cayo E.H. (2012). Sensoring fusion data from the optic and acoustic emissions of electric arcs in the GMAW-S process for welding quality assessment. Sensors.

[19] Zhao J., Sheng H., Zhou X. Study on the application of acoustic emission testing technique in monitoring 16Mn steel welding defects. Proceedings of the 2016 IEEE International Conference on Advanced Mechatronic Systems.

[20] Ancona A., Spagnolo V., Lugarà P.M., Ferrara M. (2001). Optical sensor for real-time monitoring of CO_2_ laser welding process. Appl. Opt..

[21] Sibillano T., Ancona A., Berardi V., Lugarà M.P. (2009). A real-time spectroscopic sensor for monitoring laser welding processes. Sensors.

[22] Zhang Z., Yu H., Lv N., Chen S. (2013). Real-time defect detection in pulsed GTAW of Al alloys through on-line spectroscopy. J. Mater. Process. Technol..

[23] Alfaro S.C.A., Díaz F.F. (2010). Exploring infrared sensoring for real time welding defects monitoring in GTAW. Sensors.

[24] Brock C., Tenner F., Klämpfl F., Hohenstein R., Schmidt M. (2013). Detection of weld defects by high speed imaging of the vapor plume. Phys. Proced..

[25] Wang Z. (2014). Monitoring of GMAW weld pool from the reflected laser lines for real-time control. IEEE Trans. Ind. Inform..

[26] Emilio P.L.J., Mauricio S.T.M.J., Crisostomo A.A.S. (2016). Real-time measurement of width and height of weld beads in GMAW processes. Sensors.

[27] Zhang G., Yan Z., Lin L. (2006). Reconstructing a three-dimensional P-GMAW weld pool shape from a two-dimensional visual image. Meas. Sci. Technol..

[28] Jiang C., Zhang F., Wang Z. (2017). Image processing of aluminum alloy weld pool for robotic VPPAW based on visual sensing. IEEE Access..

[29] Liu Z., Wu C., Gao J. (2013). Vision-based observation of keyhole geometry in plasma arc welding. Int. J. Therm. Sci..

[30] Comas T.F., Diao C., Ding J., Williams S., Zhao Y. (2017). A passive imaging system for geometry measurement for the plasma arc welding process. IEEE Trans. Ind. Electron..

[31] Zhang G., Wu C., Liu X. (2015). Single vision system for simultaneous observation of keyhole and weld pool in plasma arc welding. J. Mater. Process. Technol..

[32] Bardin F., Cobo A., Lopezhiguera J.M., Collin O., Aubry P., Dubois T., Högström M., Nylen P., Jonsson P., Jones J.D.C. (2005). Optical techniques for real-time penetration monitoring for laser welding. Appl. Opt..

[33] Luo M., Shin Y.C. (2015). Vision-based weld pool boundary extraction and width measurement during keyhole fiber laser welding. Opt. Lasers Eng..

[34] Harooni M., Carlson B., Kovacevic R. (2014). Detection of defects in laser welding of AZ31B magnesium alloy in zero-gap lap joint configuration by a real-time spectroscopic analysis. Opt. Lasers Eng..

[35] You D., Gao X., Katayama S. (2015). A novel stability quantification for disk laser welding by using frequency correlation coefficient between multiple-optics signals. IEEE/ASME Trans. Mech..

[36] You D., Gao X., Katayama S. (2015). WPD-PCA-based laser welding process monitoring and defects diagnosis by using FNN and SVM. IEEE Trans. Ind. Electron..

[37] You D., Gao X., Katayama S. (2014). Multisensor fusion system for monitoring high-power disk laser welding using support vector machine. IEEE Trans. Ind. Inform..

[38] Bardin F., Mcbride R., Moore A., Morgan S., Williams S., Jones J.D.C., Hand D.P. Real-time temperature measurement for process monitoring of laser conduction welding. Proceedings of the 23rd International Congress on Applications of Laser and Electro-Optics.

[39] Richardson R.W., Gutow D.A., Anderson R.A., Farson D.F. (1984). Coaxial arc weld pool viewing for process monitoring and control. Weld. J..

[40] Pietrzak K.A., Packer S.M. (1994). Vision-based weld pool width control. J. Eng. Ind..

[41] Kovacevic R., Zhang Y.M., Ruan S. (1995). Sensing and control of weld pool geometry for automated GTA welding. J. Eng. Ind..

[42] Fan C., Lv F.L., Chen S. (2009). Visual sensing and penetration control in aluminum alloy pulsed GTA welding. Int. J. Adv. Manuf. Technol..

[43] Zhang W.J., Liu Y.K., Zhang Y.M. Real-time measurement of the weld pool surface in GTAW process. Proceedings of the 2013 IEEE International Conference on Instrumentation and Measurement Technology.

[44] Wang Z. (2015). An Imaging and measurement system for robust reconstruction of weld pool during arc welding. IEEE Trans. Ind. Electron..

[45] Wang X.W. (2015). Three-dimensional vision applications in GTAW process modeling and control. Int. J. Adv. Manuf. Technol..

[46] Chen B., Wang J., Chen S. (2010). Prediction of pulsed GTAW penetration status based on BP neural network and D-S evidence theory information fusion. Int. J. Adv. Manuf. Technol..

[47] Watanabe M., Nayar S. Telecentric optics for computational vision. Proceedings of the 4th European Conference on Computer Vision.

[48] Harwood D., Subbarao M., Hakalahti H., Davis L.S. (1987). A new class of edge-preserving smoothing filters. Pattern Recognit. Lett..

[49] Otsu N. (1979). A Threshold selection method from gray-Level histograms. IEEE Trans. Syst. Man Cybern..

